# Response of Microbial Communities and Their Metabolic Functions to Drying–Rewetting Stress in a Temperate Forest Soil

**DOI:** 10.3390/microorganisms7050129

**Published:** 2019-05-13

**Authors:** Dong Liu, Katharina M. Keiblinger, Sonja Leitner, Uwe Wegner, Michael Zimmermann, Stephan Fuchs, Christian Lassek, Katharina Riedel, Sophie Zechmeister-Boltenstern

**Affiliations:** 1 Institute of Soil Research, Department of Forest and Soil Sciences, University of Natural Resources and Life Sciences Vienna (BOKU), Peter Jordan-Straße 82, 1190 Vienna, Austria; liudongc@mail.kib.ac.cn (D.L.); s.leitner@cgiar.org (S.L.); michael.zimmermann@blw.admin.ch (M.Z.); sophie.zechmeister@boku.ac.at (S.Z.-B.); 2 Key Laboratory for Plant Diversity and Biogeography of East Asia, Kunming Institute of Botany, Chinese Academy of Sciences, Kunming 650201, China; 3 International Livestock Research Institute (ILRI), Mazingira Centre for Environmental Research and Education, Box 30709, Nairobi 00100, Kenya; 4 Institute of Microbiology, University of Greifswald, Friedrich-Ludwig-Jahn-Straße 15, 17489 Greifswald, Germany; wegner@ipk-gatersleben.de (U.W.); fuchss@rki.de (S.F.); chrislassek@web.de (C.L.); riedela@uni-greifswald.de (K.R.); 5 Leibniz Institute of Plant Genetics and Crop Plant Research (IPK), Correnstraße 3, 06466, Gatersleben, Germany; 6 Swiss Federal Office for Agriculture, Mattenhofstrasse 5, 3007 Bern, Switzerland

**Keywords:** soil proteins, microbial diversity, drying–rewetting, microbial function

## Abstract

Global climate change is predicted to alter drought–precipitation patterns, which will likely affect soil microbial communities and their functions, ultimately shifting microbially-mediated biogeochemical cycles. The present study aims to investigate the simultaneous variation of microbial community compositions and functions in response to drought and following rewetting events, using a soil metaproteomics approach. For this, an established field experiment located in an Austrian forest with two levels (moderate and severe stress) of precipitation manipulation was evaluated. The results showed that fungi were more strongly influenced by drying and rewetting (DRW) than bacteria, and that there was a drastic shift in the fungal community towards a more Ascomycota-dominated community. In terms of functional responses, a larger number of proteins and a higher functional diversity were observed in both moderate and severe DRW treatments compared to the control. Furthermore, in both DRW treatments a rise in proteins assigned to “translation, ribosomal structure, and biogenesis” and “protein synthesis” suggests a boost in microbial cell growth after rewetting. We also found that the changes within intracellular functions were associated to specific phyla, indicating that responses of microbial communities to DRW primarily shifted microbial functions. Microbial communities seem to respond to different levels of DRW stress by changing their functional potential, which may feed back to biogeochemical cycles.

## 1. Introduction

In the upcoming decades, the frequency and intensity of extreme climatic events, like severe droughts and heavy rainfalls, will likely increase in many forested regions of the world [1] due to global climate change [2]. Extended periods of water limitation have been shown to reduce soil nutrient availability by inhibiting diffusion [3] and disconnecting microorganisms and plant roots from their substrates [4,5]. Furthermore, for soil microorganisms, drying and rewetting makes substantial amounts of C and N vulnerable to loss through the reallocation of resources from growth pathways to producing protective molecules, which can easily be leached or respired upon rewetting of soils [6]. Rewetting of dry soils usually leads to a pulse of C and N mineralization with a follow-up increase in carbon dioxide (CO_2_) emission (termed the “Birch Effect”) [7]. This phenomenon of enhanced CO_2_ emission after a rewetting event is suggested to be driven by both physical outgassing of CO_2_ as the water front moves through the soil, and also by the microbial mineralization of easily-available C substrates, even of microbial origin [8,9,10]. Drying induces dormancy in microorganisms that are hydrologically disconnected from their substrates (i.e., starving), reducing microbial activity and nutrient uptake [11]. At the same time, extracellular enzymes continue hydrolyzing and oxidizing organic matter well after microbial activity has slowed down, leading to an accumulation of easily-available C and N that is not immobilized by microorganisms or plant roots [12]. Upon rewetting, this labile C and N is mobilized [13] and triggers a boost in microbial activity and growth, leading to elevated respiration and soil CO_2_ emissions, even days or weeks after the rewetting event [8,11,14]. In addition to implications for biogeochemical cycling, drying stress can furthermore lead to shifts in the abundance and phylogenetic diversity of microbes [15]. In view of global climate change and the stability of forest ecosystems to DRW stress, it is important to evaluate how forest soil microorganisms respond to more frequent and longer periods of drought by the ability to survive drying and rewetting (resistance) and their capacity to return to the original functional or populational status (resilience) [16]. Due to differences in stress resistance and resilience between different microbial taxa, frequent DRW events and the associated (rapid) changes in soil moisture and substrate availability can trigger microbial population changes that likely translate into functional changes. Concerning their life strategy, microbes can be classified as either copiotrophic (fast growing, less stress resistant) or oligotrophic (slow growing, more stress resistant). Copiotrophic phyla are considered to be more competitive and to have higher resilience/survival chances during frequent drying–rewetting events because they grow fast under increased nutrient availability (as occurs during a rewetting pulse after prolonged drying) [17,18]. In contrast, oligotrophic microorganisms with a slower cell growth are usually more resistant to drought because they have more stable cell walls [19]. 

In the past, research on the influence of soil water conditions on soil microbial communities has mainly focused on: (i) microbial-physiological adaptations [6,20,21]; (ii) changes in microbial diversity assessed by DNA-based approaches (e.g., by quantifying the relative abundances of 16S rRNA genes [19] or by high-throughput sequencing [18,22]); and (iii) changes in microbial function by evaluating abundances of genes involved in C, N, and P cycles [23,24]. However, these approaches can be biased towards a limited group of microbes (e.g., by the choice of primers) and/or lack the capability of linking microbial diversity with functions related to biogeochemical cycles. 

Therefore, to assess the influence of DRW on soil biogeochemical functions mediated by changes in soil microbial diversity, an approach that can link phylogeny with function is required. With the identification of proteins that can be assigned to specific functions and phylogenetic origin, metaproteomics is a powerful tool that helps to link both structure and function of complex environmental microbial consortia [25,26]. In the present study, we used a metaproteomics approach to evaluate the impact of moderate and severe drying and rewetting cycles (two magnitudes of drought length and rewetting intensities) on the structure and function of soil microbial communities. 

We hypothesized (i) that DRW stress would induce an increase in copiotrophic taxa due to the release of easily-available C sources after rewetting [27,28]. Accordingly, we hypothesized (ii) that microbial functions would shift towards energy production to meet the increased C requirement of microbial activity and growth after rewetting, and we expected these trends to be more pronounced under severe than under moderate DRW stress. Finally, we hypothesized (iii) that bacteria would be more strongly influenced by DRW stress than fungi.

## 2. Material and Methods

This study was conducted in a mature beech forest in the Rosalia Mountains, Lower Austria (47°42′15.5″ N; 16°17′54.5″ E) at the Long Term Ecological Research (LTER) site Rosalia-Lehrforst. Mean annual precipitation was 796 mm and mean annual temperature was 6.5 °C [29]. The soil can be classified as Podsolic Cambisol according to IUSS [30], with a topsoil pH of 3.8. The experimental setup comprised two levels of precipitation manipulation: moderate level (two DRW cycles consisting of four weeks of drying followed by 75 mm rewetting) and severe level (one DRW cycle of eight weeks of drying followed by 150 mm rewetting), that were compared to controls (no manipulation). Twelve plots (2 m × 2 m) were set-up in a total of four replicate plots for each treatment and control. The litter layer, that is, the partially decomposed organic material that can be readily identified (e.g., plant leaves, twigs) was carefully moved aside during sampling to avoid the mixing of litter- and soil-inhabiting microbial communities. The remaining forest floor (F- and H-horizon) and mineral soil (A-horizon) was sampled in each plot using a soil steel probe (diameter = 3 cm) to a depth of 10 cm in triplicates that were combined to give one sample per plot (real replicates, n = 4) [13]. 

Soil samples were collected on 26 June 2013, after two months of precipitation manipulation and one day after the rewetting by manual irrigation and transported to the lab on ice. The soils were sieved (<2 mm) and stored at 4 °C for nutrient and extracellular enzyme analysis. An aliquot was frozen at −80 °C. Frozen samples from each of the four plots were pooled for extraction of proteins (see Supplementary Material for more details).

Water content (WC) was determined gravimetrically by drying moist soil samples at 105 °C for 24 h. Easily-available soil nutrients—ammonium (NH_4_–N), nitrate (NO_3_–N), and phosphate (PO_4_–P)—were determined from a soil to 1 M KCl solution (1:5 w/v) on a multimode plate reader (PerkinElmer, EnSpire). Dissolved organic C (DOC) and total dissolved N (TN) were measured on a DOC/TN analyzer (Shimadzu). Microbial biomass C (C_mic_) and microbial biomass N (N_mic_) were measured by the chloroform fumigation–extraction method, as described previously by Ross [31]. Extracellular enzyme activities (cellulase, chitinase, protease, acid phosphatase, peroxidase, and phenoloxidase) were measured photometrically based on MUF/DOPA assays [32,33]. The substrates used for measuring enzymes were described previously [34,35].

In order to extract proteins from the forest soil samples, the sodium dodecyl sulfate (SDS)–phenol method was adopted, as previously described by Keiblinger et al. [35] (for more details see Supplementary File 1). Duplicate protein extractions were performed from one composite soil sample per treatment (details on sample processing and mass spectrometry are also given in Supplementary File 1). The obtained MS/MS data were searched against NCBI entries (44,828,168 entries), downloaded from the NCBI server webpage (Available online: https://www.ncbi.nlm.nih.gov/at) on 25 June 2014. The PROteomics results Pruning and Homology group ANnotation Engine (PROPHANE) workflow (Available online: https://gitlab.com/s.fuchs/) was used to assign proteins to their phylogenetic and functional groups. Protein abundances were calculated based on the normalized spectral abundance factor (NSAF) [36]. The diversity of both microbial taxa and functions was evaluated using the Shannon diversity index (H) by considering the proportion of each class/functional category in NSAF values, according to Liu et al. [37]. Analysis of variance was performed using SPSS 17.0 software. Tukey’s multiple comparison (at *p* < 0.05) was used to test for significant differences (i) of the soil moisture content (before and after rewetting) and (ii) of soil parameters among treatments (control, moderate- and severe dry-rewetting).

## 3. Results and Discussion

After the DRW event, the volumetric soil moisture was still significantly (*p* < 0.05) lower in both moderate (~21%) and severe (~18%) stress treatments compared to controls (~31%), even though both stress treatments had been irrigated with the equivalent of one month (moderate) or two months (severe) worth of rainfall (Table 1). In a previous study at the same site, it was shown that prolonged dry conditions increased soil water repellency by changing the hydraulic soil properties (due to coating of organic matter with hydrophobic compounds) [38]. Concerning soil nutrient concentrations, the treatments of both moderate and severe DRW stress led to an insignificant decline of easily-available soil nutrients, including NH_4_^+^–N, NO_3_^-^–N, and PO_4_^3-^–P concentrations (Table 1). As ecosystem processes are often controlled by C, N, or P availability, lower nutrient availability can feed back to microbial functional dynamics. However, here we found no significant effect of DRW stress on soil N and P, which can likely be attributed to the fact that these nutrients were measured in soil extracts which have been shown to introduce a number of biases (e.g., disruption of soil aggregates during shaking, leading to co-extraction of available and occluded nutrients, or ongoing mineralization during extraction and storage) [39,40,41], which have likely masked any effects of drying and rewetting on soil nutrient availability in our study. However, our data showed reduced activity of chitinase and an increase in cellobiohydrolase with DRW stress (Table 1), indicating an increase in microbial C acquisition (cellobiohydrolase) and a decrease in N acquisition and decomposition of fungal cell walls (chitinase). 

**Figure 1:**
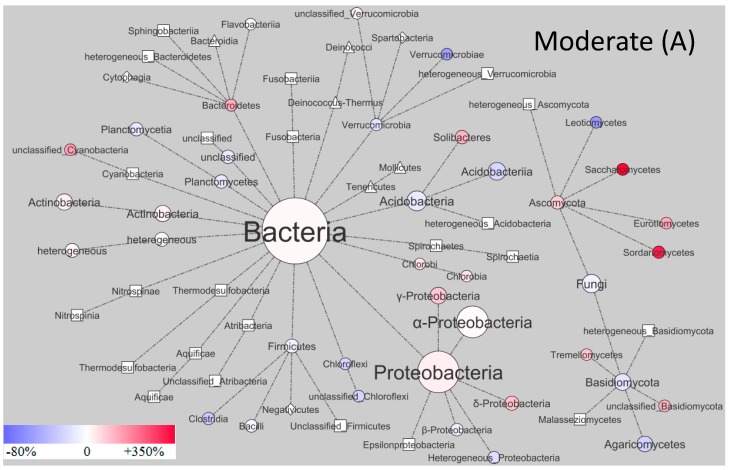

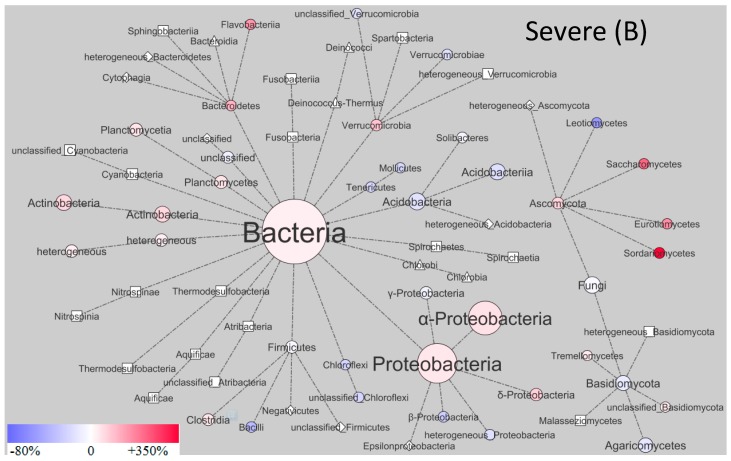
Phylogenetic network of proteins. Relative abundances calculated from the sum of the normalized spectral abundance factor (NSAFs) found for the control and drying and rewetting (DRW) plots: (**A**), moderate DRW—two consecutive cycles of four weeks of drought, then 75 mm irrigation (1 mm of precipitation refers to 1 L water m^-2^); (**B**), severe DRW—one cycle of eight weeks of drought, then 150 mm irrigation. The size of the nodes and labels represent the abundance of the corresponding taxonomic group. Labels are class-level assignments and shape-indicators are: **Diamond**, not present in control and treatments; **Triangle**, present in control but not in treatment; **Rectangle**, not present in control but in treatment; and **Circle**, present in both control and treatments. For the proteins that were present in both treatments (shown in circles) the gradient of color represents the increase (red) or decrease (blue) of the % change in the relative abundance of the treatment plots compared with control. ‘Heterogeneous’ means different microbial taxa that belong to more than one phylum/class/order. Values are means of two analytical replicates of four pooled biological replicates. Detailed changes of the percentage values in DRW treatments (as compared to controls) can be found in Supplementary Table S1.

Concerning soil microbial community composition, archaeal proteins substantially increased under both moderate (73% ± 15%; relative contribution to total microorganisms) and severe (157% ± 24%) stress compared to the control (Table 1). Many extremophiles among archaea might explain why they increase in a stressful environment (Reed et al. 2013). In response to DRW, the relative contribution of many bacterial and fungal phyla changed (Figure 1). For the bacteria, the dominant soil bacterial phylum over all treatments was Proteobacteria (>50 ± 9%), followed by Acidobacteria (~15% ± 6%) and Actinobacteria (8% ± 1%). Within the Proteobacteria, proteins that belong to the class of *α*-Proteobacteria were most prominent (~40% ± 11% of total bacteria). This is in agreement with previous studies, which reported that, *α*-Proteobacteria, Acidobacteria, and Actinobacteria are the most abundant in deciduous forests [42,43,44]. In our studied temperate forest, a large share of acidobacterial proteins was likely caused by the acidic soil conditions, because pH is one of the most prominent environmental factors determining the abundance of Acidobacteria in soils [45].

**Figure 2:**
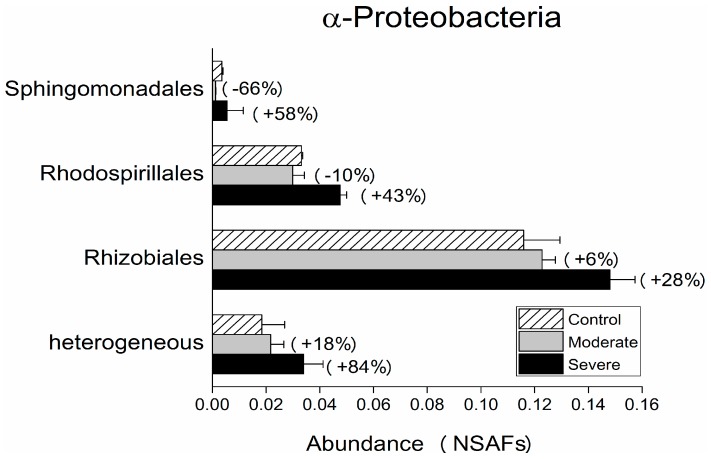
Changes in *α*-Proteobacteria abundance on the order level based on normalized spectral abundance factors (NSAFs). Moderate―two cycles of four weeks of drought, then 75 mm irrigation (1 mm of precipitation refers to 1 mm of water depth per unit area); Severe―one cycle of eight weeks of drought, then 150 mm irrigation. The percentage in brackets shows the change in unique proteins in total for each treatment. ‘Heterogeneous’ means different microbial taxa that belong to more than one phylum/class/order. Values are means ± standard deviation (n = 2 analytical replicates).

The different bacterial phyla and classes showed different responses to DRW stress. Overall, we found five phyla (Atribacteria, Thermodesulfobacteria, Nitrospinae, Fusobacteria, and Spirochaetes) that were detected only in the DRW soil but not in controls, and one phylum (Bacteroidetes) that showed a >100% change in both moderate and severe DRW treatments compared to controls. Within the most abundant phyla, an increase in Proteobacteria (Moderate, 21% ± 1%; Severe, 35% ± 14%), Actinobacteria (Moderate, 18% ± 4%; Severe, 57% ± 6%), and Bacteroidetes (Moderate, 111% ± 13%; Severe, 109% ± 12%) was observed in stressed soil. Others showed a small decline in response to re-wetting (Firmicutes −6% ± 2%, Acidobacteria −18% ± 5%, and Chloroflexi −31% ± 9%). Similar to our results, other authors have observed an increase in the abundance of Actinobacteria and a decreasing abundance of Firmicutes, Acidobacteria, and Chloroflexi after rewetting, by using DNA and RNA sequencing methods [17]. In the present study, an increase in the relative abundance of Proteobacteria under DRW was found. This can be explained by the variations at the class level, since the increase in the relative abundance of α-Proteobacteria and δ-Proteobacteria overwhelmed the decrease of β-Protebacteria (Figure 1). Furthermore, DRW resulted in an increase in Planctomycetia (35% ± 14%) in severely stressed soil (Figure 1B), which could be explained by their higher resistance against DRW stress compared to other gram-negative bacteria [42]. Within the class of *α*-Proteobacteria, DRW induced increases in the order Rhizobiales, which were the most dominant, with a relative abundance of >60% detected for both treatments. Rhizobiales increased in both treatments, which might be ascribed to their copiotrophic life strategy [46,47]. The order Rhodospirillales, also described to be copiotrophic [47], were the second-most abundant order (mean relative abundance ~20%) within the class of *α*-Proteobacteria in the present soils. However, an increase of their abundance was only observed for the severe treatment (Figure 2).

Fierer et al. [19] proposed that the oligotrophic life-strategy (slow-growing, low metabolic activity, and stable population size) might be more competitive than the copiotrophic life-strategy under DRW stress. Therefore, the abundance of Acidobacteria, which are considered to be oligotrophic [19], should decrease due to DRW stress, which was supported by our results (Figure 1). However, the response of Acidobacteria to drying–rewetting is not straight forward to explain. We would actually assume Acidobacteria to be more resistant to drought due to their slow-growing life style and high investment in the production of extracellular polymeric substances (EPS) that shield them from desiccation [48,49]. However, Banard et al. [50] reported a decrease in Acidobacteria abundance with drying and an increase with rewetting, which is in contrast to what would be suggested from the literature (instead, such a response would be expected from a copiotrophic group). In fact, the combined effect of desiccation followed by rewetting, reported by the abovementioned study, led to a similar abundance of Acidobacteria after rewetting as compared to the abundance before the onset of the drying. Furthermore, whether Acidobacteria increase or decrease in abundance is likely dependent on the severity of the stress, the availability of organic matter, and pH [42]. In addition, this supports the reasoning for site-specific changes in Acidobacteria abundance, as reported by Zhou et al. [17].

Comparing microbial community structures that changed in both moderate and severe treatments, we found that copiotrophs were more prevalent than oligotrophs in stressed plots. Copiotrophic taxa, such as Bacteroidetes, Actinobacteria *α*-, and *δ*-Proteobacteria, increased in relative abundance (Figure 1), indicating that those taxa, generally exhibiting relatively rapid growth rates and/or stimulated ribosomal synthesis [50], are favored during rewetting. As bacteria are typically more sensitive than fungi to moisture availability [5], we expected stronger fluctuations in bacterial abundances would be found in the severe DRW treatment compared to the moderate treatment. However, despite differences in the responses of individual bacterial groups to moisture availability, the coefficient of variation was similar within the treatments (0.008–0.54, moderate stress; 0.05–0.62, severe stress), and lower than the variability between treatments (0.11–1.13). A possible explanation for the similar magnitude of bacterial community changes between moderate and severe DRW may be that, i) volumetric soil moisture after rewetting was similar (moderate, ~21%; severe, ~18%); ii) the intensity of our rainfall manipulation regime was still too weak to trigger huge changes in bacterial communities, as compared to microbial long-term evolutionary climate-resistant ability (responses of bacteria at the community level are surprisingly similar across climates) [5]; and iii) during extreme precipitation events, a stronger stability lies in soil microbial community structure than in microbial activity [51].

Contrary to our expectations (hypothesis iii), fungi were more strongly influenced by DRW stress than bacteria (Figure 1). This is similar to previous works showing that fungi tend to exhibit higher population turnover [52] and increased biomass [53] in response to rewetting. Of the fungal phyla, both control and DRW treatments were dominated by Basidiomycota (>75% ± 16%) and Ascomycota (~20% ± 7%). Within the Basidiomycota, proteins that belong to the class of Agaricomycetes were most prominent (~70% of total fungi). This is in accordance with the findings on the soil fungal community response to rewetting, based on 28S rRNA gene pyrosequencing [54]. Furthermore, we found a shift from Basidiomycota to Ascomycota with DRW, which resulted in an increased ratio of Asco-/Basidomycota (Table 1). The fungal community, more specifically the increasing abundance of Ascomycota, points towards the mobilization of easily-available nutrients after rewetting, as this phylum generally favors simple C substrates [28]. This is in line with the increased cellobiohydrolase activity we observed after rewetting. Furthermore, within the phylum Ascomycota, an increase in Sordariomycetes (Moderate, 314% ± 69%; Severe, 348% ± 84%), Saccharomycetes (Moderate, 343% ± 54%; Severe, 240% ± 47%), and Eurotimycetes (Moderate, 133% ± 22%; Severe, 40% ± 9%) was observed. The DRW stress likely favored the phylum Ascomycota because it exhibits a fast-growing copiotrophic life-strategy. With regards to the Basidiomycota, Leotiomycetes and Agaricomycetes declined by approximately 72% ± 16% and 21% ± 5% in response to DRW stress. Similar to our results, increases in Sordariomycetes and Saccharomycetes abundance and a decreasing abundance of Agariomycetes were also observed in response to rewetting [54]. Within the Basidiomycota, the class of Agaricomycetes, dominated by the order Agaricales (which includes a number of ectomycorhizal species), decreased in DRW plots (Moderate, 27% ± 5%; Severe, 16% ± 3%). Besides this, we observed that all fungal classes displayed a similar change in response to moderate and severe DRW treatments, which could be linked to a larger resistance of the fungal community composition to strong alterations in soil moisture [55,56,57,58]. Taken together, we observed a higher relative variation of distribution (measured as coefficient of deviation (CV) = SD/mean) among fungal phyla (CV, 0.16–0.74) than among bacterial phyla (CV, 0.14–0.46).

**Figure 3:**
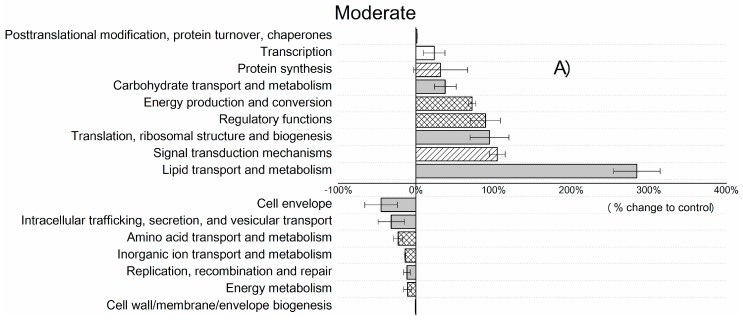

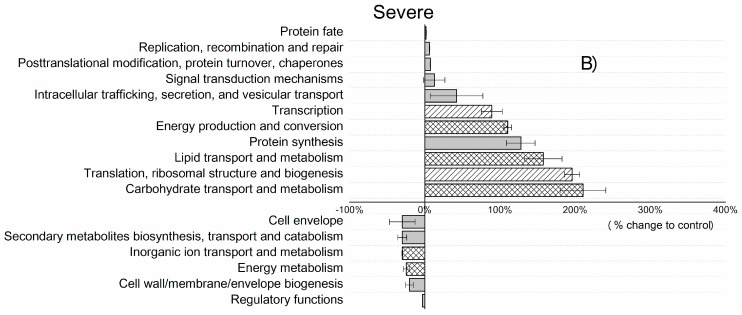
Functional distribution of microbial proteins based on clusters of COG/KOG (Clusters of Orthologous Groups of Proteins/Eukaryotic Ortholog Groups) main/sub role. For COG and KOG functional categories, protein functions are screened only from the origin of bacteria and fungi, respectively, but not for archaea, as such data are so far not available. Relative abundances calculated from the sum of normalized spectral abundance factor (NSAFs) found in the control and DRW plots: (**A**) moderate DRW treatment (**B**) severe DRW. Bars are means of two biological replicates and show the % change in relative abundance of the treatment plots compared with the control. In main functional classifications, cellular processes are plain, metabolisms are crosshatched, and information storage and processing are striped from bottom left to top right.

In addition to phylogenetic changes, DRW also influenced the functional structure/categories of the microbial community as well as proteins involved in lipid transportation, ribosomal structure and carbohydrate transportation. Specifically, moderate DRW stress induced a ~300% increase in proteins assigned to the functions “lipid transport and metabolism” (Figure 3A), suggesting that a number of proteins related to metabolism and lipid transport were activated under moderate DRW stress. This might explain the increased total microbial abundance, because lipid transport and metabolism are related to the synthesis of phospholipids, which are the main components of microbial cell membranes. This supports the notion that some microbes respond to rewetting with increased growth and proliferation, which is in line with the findings of Nielsen and Ball [59], who demonstrated that a medium precipitation event activated microbial cell growth. Furthermore, we suggest that in the present study, the increased expression levels of ribosomal proteins (Table 1) and proteins assigned to the functions “translation, ribosomal structure, biogenesis” and “protein synthesis” likely indicate pronounced microbial cell growth in moderately and severely stressed DRW soils (Figure 3).

Generally, rewetting triggers a strong increase in CO_2_ emissions, which has been attributed to both physical outgassing of soil CO_2_ and enhanced microbial activity and growth [60]. In the present study, the latter explanation of the Birch effect is supported by an increase in proteins linked to the intracellular functions of “carbohydrate transport and metabolism” and “energy production and conversion” in the severe DRW treatment (Figure 3B).

**Figure 4:**
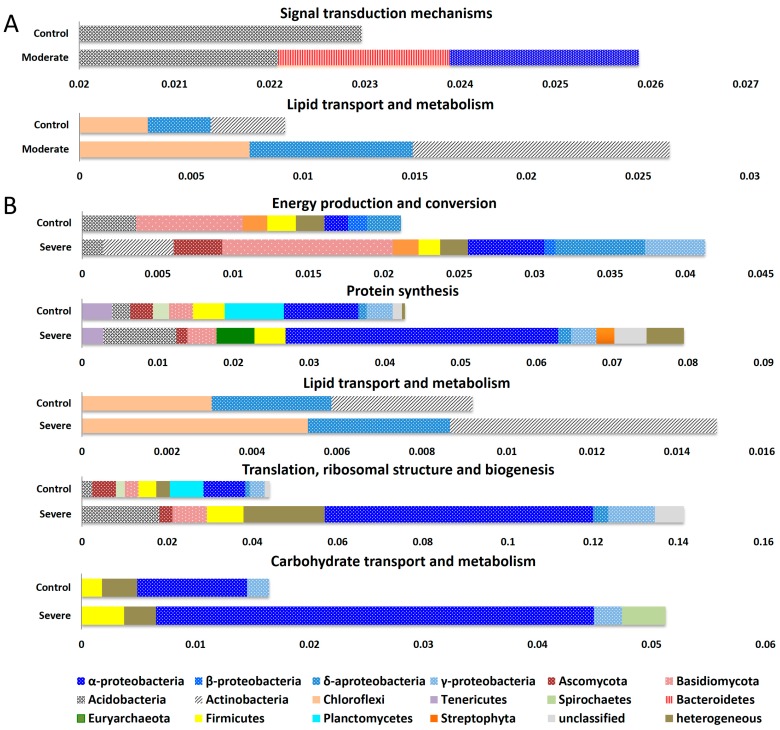
Microbial community functions in drying and rewetting (DRW) treatments that were different from controls (>100%) assigned to their taxonomic origin. (**A**) moderate DRW; (**B**) severe DRW. Microbial community functionality is based on normalized spectral abundance factors (NSAFs) of microbial proteins. Values on the x-axes are NSAF values of microbial functions assigned to their phylogentetic origin at class-level. To indicate their origin on the phylum level, shadings were included: blue dotted bars indicate Proteobacteria, red dotted bars indicate Fungi, Acidobacteria are crosshatched, Actinobacteria are striped from bottom left to top right, Bacteroidetes are vertical red lines, and heterogenous are plain.

The metaproteomics approach enabled us to link intracellular functions to their phylogenetic origin (Figure 4), where we focused on functions that changed in their relative abundance by more than 100% compared to controls. The strong increase in the lipid transport and metabolism function was related to the increased expression by Actinobacteria, and the strong increase in the signal transduction mechanisms was directly related to expression by α-Proteobacteria and Bacteroidetes (Figure 4A). The increased lipid transport and metabolism in moderate DRW soil can be an indirect reflection of decreased availability of simple C substrates [61] and reduced degradation of the cell wall and membranes from other clades [62]. Increase in the function of lipid transport and metabolism in both DRW treatments was related to higher *δ*-Proteobacteria and Actinobacteria abundance (Figure 4B); although Chloroflexi abundance declined, their contribution to enhanced lipid transport and metabolism was observed in both treatments (Figure 4). Protein function translation and ribosomal structure and biogenesis were described as indicators for copiotrophs [63], which were enhanced more than 90% and 190% in moderate and severe DRW treatments, respectively. In severe DRW, a ~100% increase in proteins assigned to energy production and conversion was directly associated with a higher abundance of proteobacterial proteins, as well as the abundance of Ascomycota (Figure 4B). The functions of carbohydrate transport and metabolism, which increased by approximately 200%, were mainly associated with an increase in the abundance of *α*-Proteobacteria (Figure 4B). Concisely, the short-term DRW might cause a quick mineralization of C and availability of easily degradable C-fraction [8]. 

In terms of the category that has an over one-fold change (comparing treatments (moderate/severe) and control), there were four functional categories which showed >100% change from the total of 16 (Figure 3), while for microbial abundance, only one phylum (Bacteroidetes) showed >100% variation among the 25 microbial phyla (Figure 1). Additionally, the average percentage change was also higher in function (42%) than in microbial abundance (14% in phylum; 38% in class). Taken together, soil microbes undergoing DRW stress exhibited stronger changes in function (CV, 0.45–1.55) than in microbial abundance (CV, 0.11–1.13). Copiotrophic phyla became more prevalent and increased more strongly in severe than in moderate DRW. Domains of functional categories varied distinctly in different DRW intensities. In the severe DRW treatment, the copiotrophic *α*-Proteobacteria was the main phylum responsible for carbohydrate transport and metabolism. In contrast, in the moderate DRW treatment, the copiotrophic phylum Actinobacteria was highly involved in lipid transport and metabolism.

## 4. Conclusions

Our study shows that adaptation of soil microorganisms to extreme soil desiccation and rapid rewetting is expressed through distinct changes in (i) fungal community structure, (ii) microbial intracellular functions and, to a smaller extent, (iii) different microbial life strategies. The response was related to changes in C mineralization. In particular, there was a strong shift towards a more Ascomycota-dominated fungal community, who are suggested to act as primary decomposers of easily-available C sources, which are increasingly available following the rewetting of dry soil. An increase in protein functions assigned to carbohydrate transport and energy conversion as a response to drought and rewetting was observed, as well as a trend for higher abundance of copiotrophic taxa that, generally, utilize more easily-available C to produce energy. These results support the claim that changes in precipitation regimes affect the soil microbial community and its functions, with potential effects on C budgets and nutrient cycling on a large scale. 

## Figures and Tables

**Table 1:** Water content, physicochemical properties, microbial biomass, extracellular enzymes, and Shannon indices of soil microbial taxa and functional diversity. Data show mean and standard errors (n = 4); significant differences according to Tukey’s multiple comparison test (*p* < 0.05) are indicated with different lowercase letters (* marked on the corresponding soil indices).

	Control	Moderate	Severe
* Water content %_w/w_	30.9 ± 2.6 ^a^	21.3 ± 1.7 ^b^	17.7 ± 2.1 ^b^
NH_4_^+^–N (µg g^−1^)	2.72 ± 0.27	2.54 ± 0.26	2.48 ± 0.33
NO_3_^−^–N (µg g^−1^)	4.32 ± 0.44	4.11 ± 0.74	3.63 ± 0.25
PO_4_^3−^–P (µg g^−1^)	1.56 ± 0.55	1.31 ± 0.36	1.10 ± 0.49
Dissolved organic carbon (µg g^−1^)	197 ± 18	187 ± 53	212 ± 61
Total dissolved N (µg g^-1^)	25.7 ±1.9	25.8 ± 6.35	26.7 ± 9.5
Microbial biomass carbon (mg g^−1^)	1.21 ±0.24	0.99 ± 0.10	0.96 ± 0.19
Microbial biomass nitrogen (mg g^−1^)	0.12 ± 0.04	0.10 ± 0.01	0.09 ± 0.02
Cmic/Nmic ratio	10.3 ± 1.5	10.1 ± 1.38	10.2 ± 0.8
* Cellobiohydrolase (nmol g^−1^ min^−1^)	4.0 ± 2.1 ^b^	3.4 ± 1.6 ^b^	7.1 ± 1.1 ^a^
* Chitinase (nmol g^−1^ min^−1^)	13.1 ± 1.8 ^a^	9.4 ± 2.4 ^b^	8.9 ± 3.2 ^b^
Phosphatase (nmol g^−1^ min^−1^)	54.8 ± 10.3	35.2 ± 6.2	42.5 ± 11.7
Protease (nmol g^−1^ min^−1^)	2.8 ± 0.2	2.1 ± 0.1	2.1 ± 0.2
Phenoloxidase (nmol g^−1^ min^−1^)	14.6 ± 7.1	8.9 ± 3.1	10.8 ± 4.4
Peroxidase (nmol g^−1^ min^−1^)	84.6 ± 46.4	123.4 ± 55.9	204.6 ± 118.8
Archaeal proteins (NSAFs)	0.0017	0.0030	0.0045
Expressed ribosomal proteins	7	38	23
Ascomycota/Basidiomycota (NSAFs ratio)	0.16	0.34	0.31
Shannon ^a^-taxa diversity ^b^	2.73	2.79	2.51
Shannon-functional diversity ^c^	5.39	5.71	5.62

^a^ Shannon diversity index (H) retrieved with NSAF values; ^b^ Taxa diversity, bacterial taxa at class-level; ^c^ Functional diversity, microbial proteins based on cluster of orthologous groups.

## Supplementary Materials

The supplementary materials are available online at https://www.mdpi.com/2076-2607/7/5/129/s1.
