# Supplementary material for: Response of Microbial Communities and Their Metabolic Functions to Drying–Rewetting Stress in a Temperate Forest Soil
Source: Microorganisms. 2019 May 13;7(5):129. doi: 10.3390/microorganisms7050129 (PMC6560457; doi:10.3390/microorganisms7050129)
Supplement: Supplementary file 1 [file microorganisms-07-00129-s001.pdf]

microorganisms

Supplementary Material

Response of Microbial Communities and Their Metabolic Functions to Drying–Rewetting Stress in a Temperate Forest Soil

Dong Liu [1,2,†], Katharina M. Keiblinger [1,*,†], Sonja Leitner [1,5], Uwe Wegner [3,6], Michael Zimmermann [1,7], Stephan Fuchs [3,4], Christian Lassek [3], Katharina Riedel [3] and Sophie Zechmeister-Boltenstern [1]

[1] Institute of Soil Research, Department of Forest and Soil Sciences, University of Natural Resources and Life Sciences Vienna (BOKU), Peter Jordan-Straße 82, 1190 Vienna, Austria; liudongc@mail.kib.ac.cn (D.L.); s.leitner@cgiar.org (S.L.); michael.zimmermann@blw.admin.ch (M.Z.); sophie.zechmeister@boku.ac.at (S.Z.-B.)
[2] Key Laboratory for Plant Diversity and Biogeography of East Asia, Kunming Institute of Botany, Chinese Academy of Sciences, Kunming 650201, China
[3] Institute of Microbiology, University of Greifswald, Friedrich-Ludwig-Jahn-Straße 15, 17489 Greifswald, Germany; wegner@ipk-gatersleben.de (U.W.); fuchss@rki.de (S.F.); chrislassek@web.de (C.L.); riedela@uni-greifswald.de (K.R.)
[4] present address: Robert-Koch-Institute, Nosocomial Pathogens and Antibiotic Resistance, Burgstraße 37, 38855 Wernigerode, Germany
[5] International Livestock Research Institute (ILRI), Mazingira Centre for Environmental Research and Education, Box 30709, 00100 Nairobi, Kenya
[6] Leibniz Institute of Plant Genetics and Crop Plant Research (IPK), Correnstraße 3, 06466, Gatersleben, Saxony-Anhalt, Germany
[7] Swiss Federal Office for Agriculture, Mattenhofstrasse 5, 3007 Bern, Switzerland
[†] These authors contributed equally to this study
[*] Corresponding author: katharina.keiblinger@boku.ac.at; Phone: +43 1 47654 91141

1. Full methodological descriptions

1.1. Experimental design

To explore the response of microbes to drying-rewetting, we chose a 2-month time interval (from April to June, 2013) from an established field experiment with irrigation manipulation. Two types of artificial-simulated drying-rewetting stress were — 2 cycles of 4 weeks drought, then 75 mm irrigation (moderate treatment) and 1 cycle of 8 weeks drought, then 150 mm irrigation (severe treatment). Specifically, for each treatment and controls, four replicate plots were set-up. Each plot has a size of 2 x 2 m. We established our sampling plots >2 m distant from trees in order to minimize boundary effects. To simulate drought, 4 x 4 m roofs were made out of transparent acrylic panels and wooden scaffolding were mounted 1.2m above the artificial plots to exclude precipitation. To simulate various density rainfalls, rewetting was performed through an automated irrigation system after each drought period. To prevent lateral water flow on plots located on the slope, we dug trenches above stressed plots.

1.2. Protein extraction

Protein extraction was done according to the method described by Keiblinger et al., (2012) on pooled samples. Cell disruption and purification were performed by mixing soil samples with 10% (w/w) polyvinylpolypyrrolidone (PVPP), and grounding in liquid nitrogen. The disruption of soil aggregates was performed by ultra-sonicating the sample on ice for 1 min (10% energy, continuous mode), followed by shaking at 150 rpm and 20 °C (30 min). Proteins extraction was performed by using a phenol SDS buffer (1:1 (v:v) SDS-phenol buffer — 50 mM Tris, 1% SDS (pH 7.5) + phenol (pH 8.0)). The purified phenol phases were combined and proteins were precipitated with ammonium

acetate by centrifugation 10640 g for 20 min at 4 °C. The pellets were washed with 100% pre-chilled acetone by vortexing and a further centrifugation step. To remove substances which interfere with further processing (protein digestion, peptide separation and MS analysis), we precipitated the samples with the 5-fold amount of 0.1 M ammonium acetate in methanol over night at −20 °C. Before polyacrylamide gel electrophoresis (Benndorf et al., 2007), the protein pellets were resuspended in a maximum of 1 ml 0.5 M TEAB buffer containing 10 mM dithiothreiol (DDT), 6 M urea and 1 M thiourea by vortexing and gentle shaking over night at 4 °C (Keiblinger et al., 2012). The resulting supernatant was used for further processing. Extracted proteins were loaded on SDS gels (5% polyacrylamide (stacking gel) + 12% polyacrylamide (separating gel).

1.3. Protein digestion

After electrophoresis, the obtained gel was stained with Coomassie Brilliant Blue-G-250 (Sigma-Aldrich, Steinheim, Germany) and protein lanes were cut into ~10 small pieces. Gel pieces were destained. Destaining steps were repeated as often as necessary to get colorless dices (200 mM NH_4HCO_3, 30% acetonitrile); dried in a vacuum centrifuge and the gel slices were digested by employing 2 µg ml^{-1} sequencing grade modified trypsin (Promega, reference V5111) over night at 37 °C. The resulting peptide mixtures were C-18 purified (Zip-tip, Millipore, Billerica, MA, USA) according to the indoor protocol and analysed by Liquid chromatography tandem-mass spectrometry (LC-MS/MS).

1.4. Mass Spectrometry analysis

Therefore, an Easy-nLC II (Thermo Fisher Scientific, Waltham, U.S.) was coupled to an LTQ Orbitrap Velos (Thermo Fisher Scientific, MA). Chromatographic separation of peptides was achieved using a 100 min gradient with buffer A (0.1% (v/v) acetic acid) and buffer B (99.9% (v/v) acetonitrile, 0.1% (v/v) acetic acid) and a flow rate of 300 nL/min on a self-made C18 column (Luna 3n, 100 µm i.D. × 200 mm column, Phenomenex, Aschaffenburg, Germany). The mass spectrometer was operated in data-dependent MS/MS mode using wideband activation and lock mass option for the 445.120025 ion. The resolution of the full scan in the Orbitrap analyzer was recorded at R = 60,000. After the survey scan MS/MS data were acquired for the 20 most intensive precursor ions in the linear ion trap using collision induced dissociation (CID) for fragmentation. Charge state screening was employed to select for ions doubly charged or higher and rejecting ions in single-charge state.

1.5. Data base searches, processing and validation

Raw data files were searched using Mascot (Matrix Science Version 2.4.1) against the NCBnrI database (44828168 entries) (state 25th June 2014). The following settings were selected: tryptic cleavage with a maximum of two missed cleavage sites; fragment ion tolerance: 0.50 Da (Monoisotopic) and peptide tolerance: 10.0 ppm; variable Modifications: +16 on M (Oxidation). Following filters were used: peptide probability min. 95 % as specified by the Peptide Prophet algorithm (Keller et al., 2002) (FDR <1.2%, Prophet), protein probability (min. 99 %) was assigned by the Protein Prophet algorithm (Nesvizhskii et al., 2003) (FDR< 0.4%, Prophet) and at least one unique peptide per protein. Protein Grouping Strategy was experiment-wide grouping with binary peptide-protein weights.

1.6. Assignment of data to phylogenetic and functional groups

Before assigning to functional and taxonomic classes protein groups were checked for homology. Heterogeneous groups were excluded from further analysis. Homologous protein hits obtained by the database searches were assigned to phylogenetic and functional groups and assignments were done by a newly developed perl-script based PROteomics result Pruning & Homology group ANotation Engine (PROPHANE) (Schneider et al. 2011) workflow (https://gitlab.com/s.fuchs/). Homology was checked by Prophane using MAFFT (for details view: http://nar.oxfordjournals.org/content/30/14/3059.full).

References

Schneider, T., E. Schmid, J. V. de Castro, M. Cardinale, L. Eberl, M. Grube, G. Berg, and K. Riedel, 2011. Structure and function of the symbiosis partners of the lung lichen (Lobaria pulmonaria L. Hoffm.) analyzed by metaproteomics. Proteomics. 11:2752-2756.

Keller, A., Nesvizhskii, A., Kolker, E., Aebersold, R, 2002. Empirical statistical model to estimate the accuracy of peptide identifications made by MS/MS and database search. 20:5383-5392.

Keiblinger K.M., Wilhartitz I.C., Schneider T., Roschitzki B., Schmid E., Eberl L., 2012. Soil metaproteomics — comparative evaluation of protein extraction protocols. Soil Biol Biochem, 54: 14–24.

Nesvizhskii, A., Keller, A., Kolker, E., Aebersold, R, 2003. A statistical model for identifying proteins by tandem mass spectrometry. 17: 4646-4658.

Website and Online Resources

MAFFT webpage. Available online: http://nar.oxfordjournals.org/content/30/14/3059.full.

ANotation Engine (PROPHANE) workflow webpage. Available online https://gitlab.com/s.fuchs/.

© 2019 by the authors. Submitted for possible open access publication under the terms and conditions of the Creative Commons Attribution (CC BY) license (http://creativecommons.org/licenses/by/4.0/).
